# Rare Central Venous Catheter Malpositions: A Case Series

**DOI:** 10.7759/cureus.63872

**Published:** 2024-07-04

**Authors:** Büşra Tanyıldızı

**Affiliations:** 1 Anesthesiology and Reanimation, Kastamonu Education and Research Hospital, Kastamonu, TUR

**Keywords:** central venous catheter, chest x ray, ultrasound-guided, intensive care, malposition

## Abstract

Central venous catheters are a procedure that provides vascular access, allowing the application of various clinical treatments and the measurement of some hemodynamic values. It provides access to the internal jugular vein, subclavian vein, and, femoral vein with a large-bore catheter. There are mechanical, infectious, and thromboembolic complications resulting from central venous catheter placement and care. Central venous catheter malposition is a rare catheter complication that may be encountered. The location of the central venous catheter can be evaluated with imaging techniques such as posteroanterior chest radiograph, ultrasonography, central venous catheter waveform, and transesophageal echocardiography. Five malposition cases detected by imaging after the central venous catheter procedure in our clinic are presented.

## Introduction

Central venous catheters (CVCs) are widely used to provide vascular access in clinical indications such as fluid resuscitation, drug administration, renal replacement therapy, total parenteral nutrition, and cardiac transvenous pacemaker catheterization [1]. CVC provides venous access via a CVC placed in the internal jugular vein (IJV), subclavian vein (SCV), and femoral vein [2]. The IJV on the right side is generally preferred for CVC placement. The reason is that the IJV is directly connected to the right atrium, is easily accessible, and this part of the heart is not prone to complications [2]. Sometimes, the IJV becomes difficult to access due to reasons concerning head and neck tumors or trauma, SCV is the vein of choice. The advantages of SCV are significantly low rates of both infectious and thrombotic complications [3].

While CVC placement and care have many benefits, there are also risks and side effects during or after the procedure. The most common complications include infectious complications with a frequency of 5-26%, thromboembolic complications with a frequency of 2-26%, and mechanical complications with a frequency of 5-19%. Some of the mechanical complications are arterial puncture and subsequent bleeding or hematoma, arrhythmia, hemothorax, pneumothorax, air embolism, catheter infection, guidewire misplacement, and catheter malposition [1-4]. Various studies have stated that the effectiveness and success of CVC placement increase when ultrasound guidance is used instead of relying solely on anatomical points. There are studies reporting that the USG-guided technique reduces related complications and unsuccessful punctures compared to the anatomical point for the placement of IJV, SCV, and femoral CVCs [5]. Catheter malposition is a complication, and catheter malposition rates are reported to be between 3.6% and 14% in small and medium-sized clinical studies [6]. Catheter malpositions are generally rare complications that may be encountered. In this case series presentation, catheter malpositions are explained in different cases.

This study was presented as an oral presentation at the 9th UTSAK, 18/19 March 2022, Online/ANKARA congress.

## Case presentation

Case 1

A 78-year-old woman with pneumonia, diabetes mellitus, and hypertension was admitted to the postoperative intensive care unit for careful monitoring of her hemodynamic status after incarcerated hernia surgery. On the 34th day of intensive care treatment, a 7-French triple-lumen catheter was inserted into the right SCV using the Seldinger technique under USG guidance for the purpose of changing the CVC.

**Figure 1:**
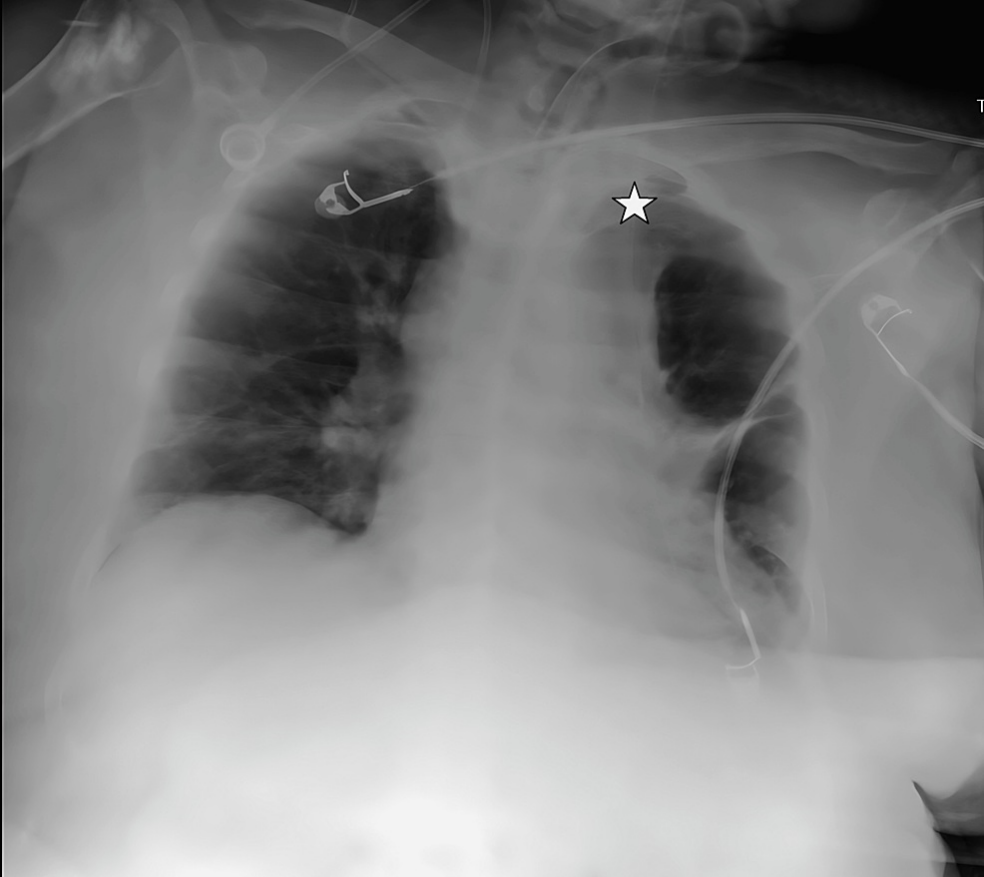
CVC transition from the right subclavian vein to the left brachiocephalic vein on chest radiograph imaging. The star symbol shows the tip of the catheter. CVC: central venous catheter

The catheter was fixed at 12 cm. Blood was taken from all three lines of the catheter. Postprocedural evaluation of posteroanterior chest radiograph imaging revealed that the catheter was placed on the opposite side of the brachiocephalic vein (Figure 1).

Case 2

**Figure 2:**
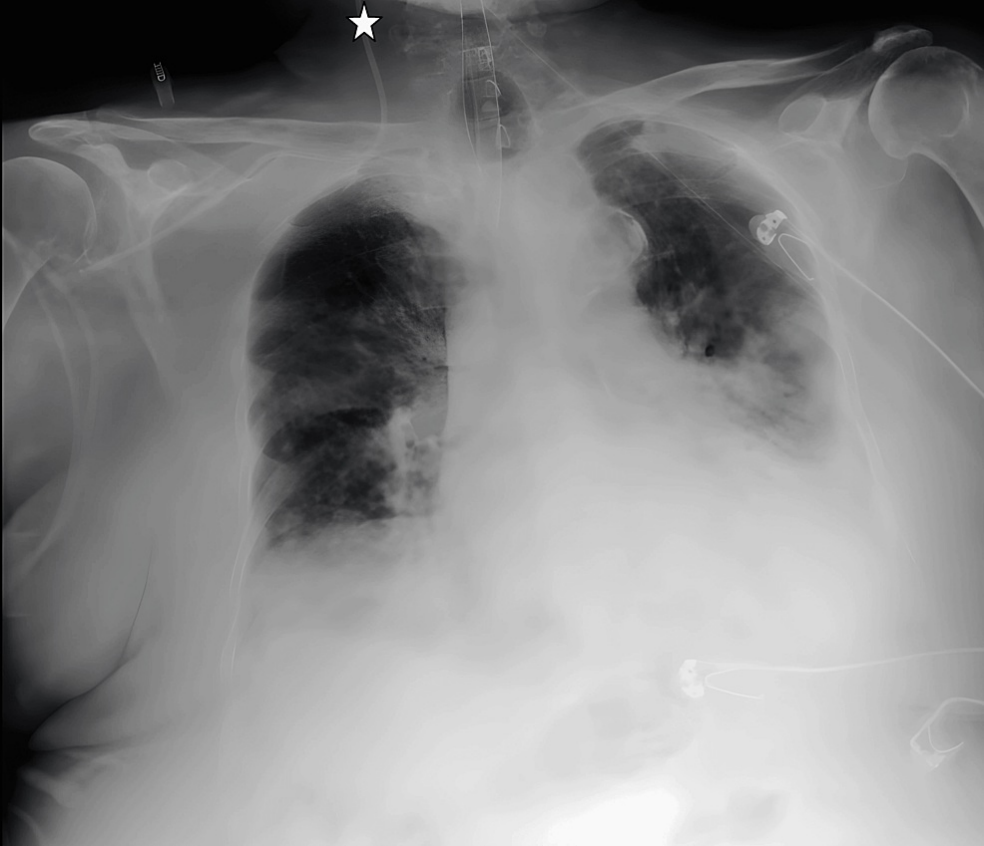
CVC transition from the right subclavian vein to the right internal jugular vein on chest radiograph imaging. The star symbol shows the tip of the catheter. CVC: central venous catheter

A 90-year-old female patient with hypertension, hypothyroidism, obesity (BMI 37), and dementia was admitted to the intensive care unit as a result of COVID-19 pneumonia. On the 23rd day of the patient, who was still hospitalized due to pneumonia, a 7-French triple lumen catheter was placed in the right SCV using the Seldinger technique, without USG guidance, for CVC replacement. The catheter was fixed at 13 cm. Blood was taken from all three lines of the catheter. Postprocedural evaluation of posteroanterior chest radiograph imaging revealed that the catheter was inserted into the right IJV (Figure 2).

Case 3

**Figure 3:**
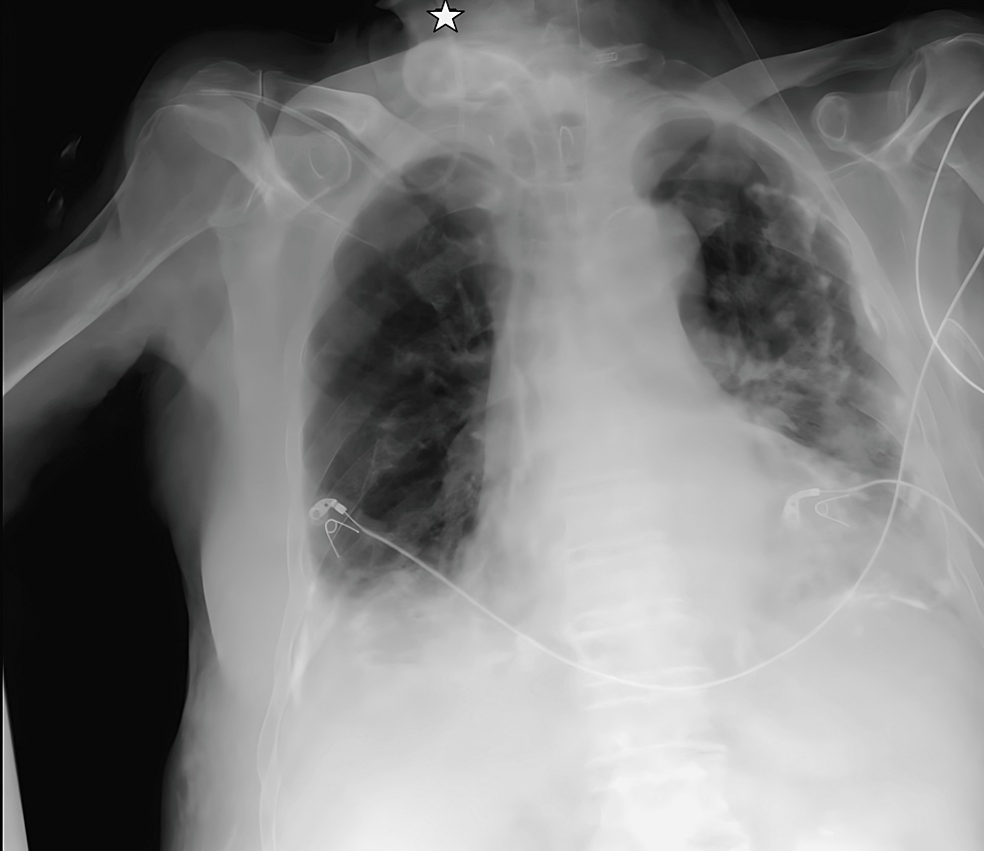
Transition from the right subclavian vein to the right internal jugular vein on chest X-ray imaging. The star symbol shows the tip of the catheter.

**Figure 4:**
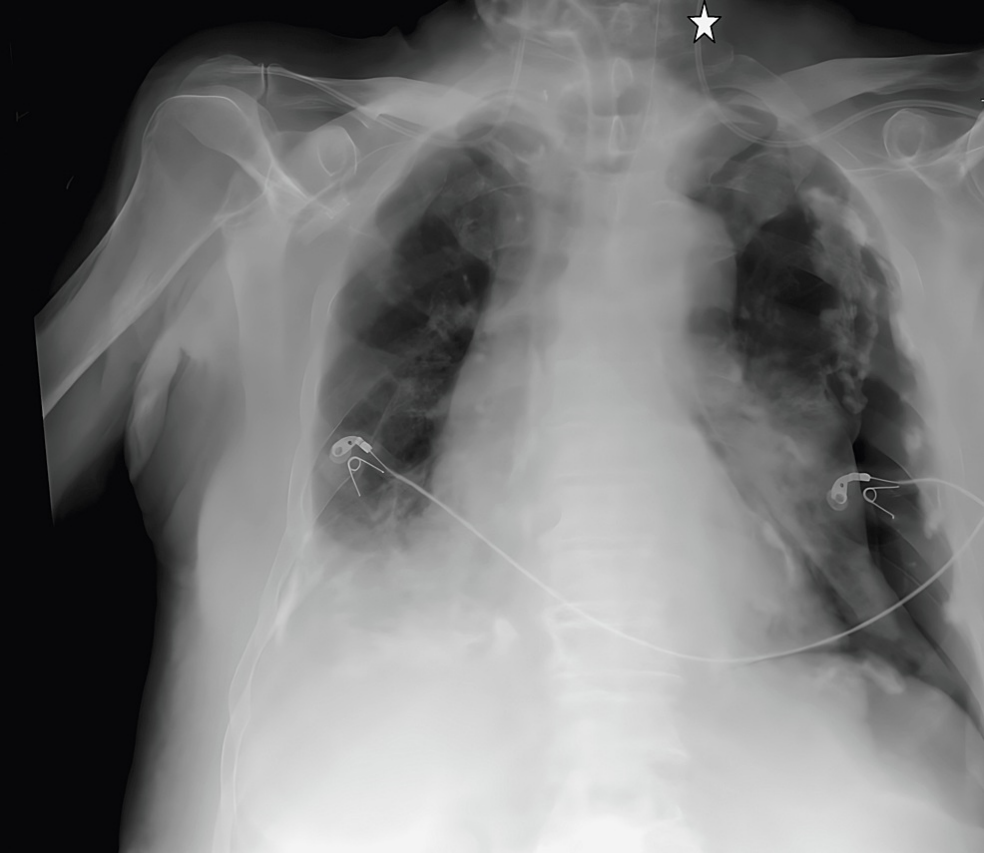
Transition from the left subclavian vein to the left internal jugular vein on chest X-ray imaging. The star symbol shows the tip of the catheter.

An 88-year-old male patient was admitted to intensive care with a diagnosis of septic shock. The patient was planned to have a CVC catheter inserted on the 27th day for inotropic treatment and high-volume fluid replacement. A 7-French triple-lumen catheter was placed in the right SCV using the Seldinger technique under USG guidance. The catheter was fixed at 12 cm. Blood was aspirated from all three lines of the catheter. When the posteroanterior chest radiograph was evaluated, the catheter was seen to move from the right SCV to the right IJV (Figure 3). The intervention was repeated from the left SCV. After the procedure, posteroanterior chest radiograph imaging was evaluated, and this time it was observed that the catheter was placed in the left IJV (Figure 4).

Case 4

**Figure 5:**
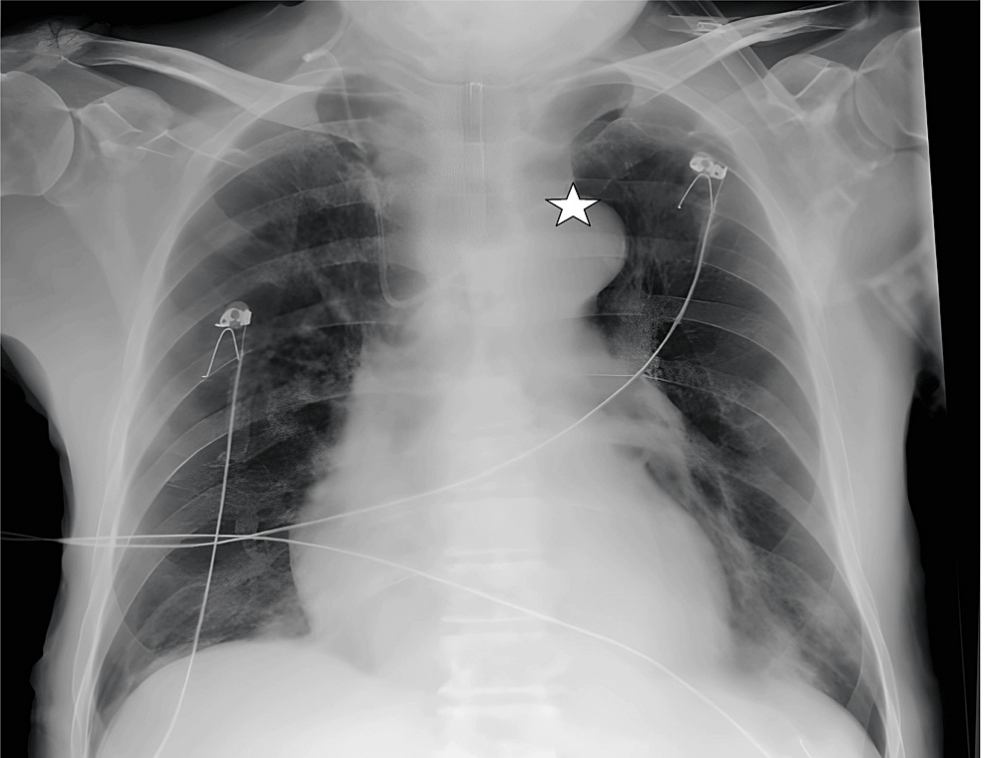
Kink of the catheter in the internal jugular vein on chest X-ray imaging, return from the right brachiocephalic vein to the left brachiocephalic vein. The star symbol shows the tip of the catheter.

A 71-year-old male patient with diabetes mellitus, coronary artery disease, and hypertension was admitted to the intensive care unit due to diabetic ketoacidosis. On the 43rd day of hospitalization, the patient developed acute renal failure and peripheral arterial circulation disorder during his treatment in the intensive care unit. A 7-French triple lumen catheter was placed in the right IJV using the Seldinger technique, without USG guidance, for CVC replacement. The catheter was fixed at 14 cm. Blood was extracted from all three lines of the catheter. During the posteroanterior chest radiograph imaging following the procedure, we observed that the catheter kinked in the right IJV and passed from the right brachiocephalic vein to the left brachiocephalic vein (Figure 5).

Case 5

**Figure 6:**
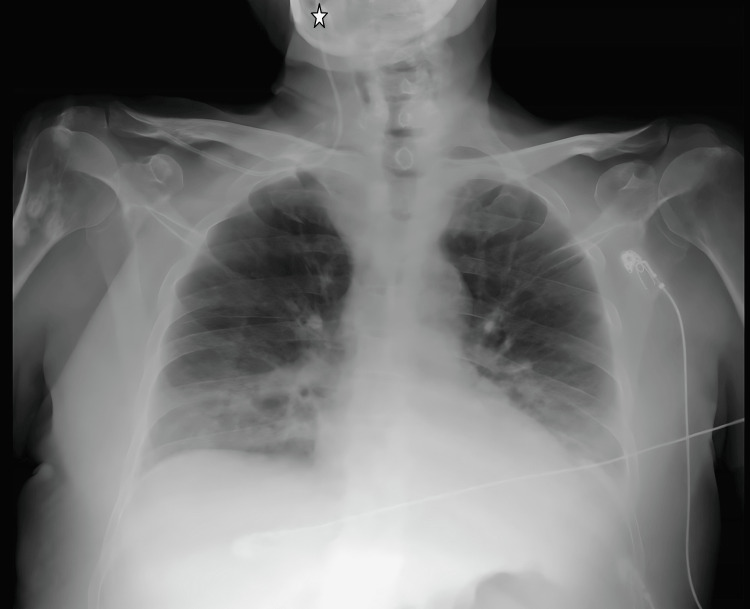
Transition of the catheter from the right subclavian vein to the right internal jugular vein on chest X-ray imaging. The star symbol shows the tip of the catheter.

A 66-year-old male patient with coronary artery disease and hypertension was admitted to the intensive care unit after cardiopulmonary resuscitation. On the 12th day of the patient, who was hospitalized due to pneumonia, a 7-French triple lumen catheter was inserted into the right SCV using the Seldinger technique, without USG guidance, for CVC replacement. The catheter was fixed at 13 cm. Blood was extracted from all three lines of the catheter. After the procedure, the posteroanterior chest radiograph imaging revealed the placement of the catheter in the right IJV (Figure 6).

## Discussion

Malposition is a complication that can be observed during central catheter placement using the blind technique. Post-procedure posteroanterior chest radiograph imaging is very important in determining the diagnosis. According to a study by De Backer and Vincent, it was found that CVC can sometimes move to unexpected areas such as the internal mammary vein, azygos vein, hemiazygos vein, upper intercostal veins, and thymic vein [7]. In the cases mentioned in this case series, the malposition is localized in the subclavian and IJVs.

During the CVC procedure, the patient should be placed in a supine position. When planning an intervention involving the upper extremity, it is recommended to provide under-shoulder support and take Trendelenburg’s position into consideration [2]. It was thought that the back support used during catheter insertion in Case 1 and Case 5 may have been inadequate. It was thought that appropriate positioning of the patient would reduce the risk of malposition as it would align the anatomical structures correctly.

Another factor that makes it difficult to insert the central catheter using a blind technique is the difficulty in determining the patient’s BMI and anatomical intervention areas [8]. In Case 2, the patient’s obesity may be the cause of malposition.

Bedel et al. stated that malposition cases can be minimized by guidewire localization guided by transesophageal echocardiography (TEE) [9]. If this technique had been used in the third case, in which the central catheter procedure was tried twice in the treatment of the patient, the same complications could have been prevented in the second attempt. It is possible that rotation towards the ipsilateral IJV is due to anatomical problems. The patient is 88 years old and age-related osteoporosis, changes in anatomical structures and vascular changes can cause this.

During the CVC placement process using the Seldinger technique, it is critical to ensure that blood can flow freely and that no resistance occurs when placing the guidewire [2]. During CVC placement, in our fourth case, there was a sticking sensation during guidewire placement.

Studies report that posteroanterior chest radiograph imaging or ultrasonography can be used to detect malposition [10,11]. Since the posteroanterior chest radiograph provides us with a 2-dimensional view, it may not always be enough to confirm the location of the catheter. A catheter accidentally placed in areas such as the internal mammary vein, vertebral vein, and mediastinum may appear in its normal course on a posteroanterior chest radiograph. There are also cases detected intraoperatively during cardiothoracic surgery [12,13].

In the mentioned cases, the confirmation of catheter placement was done using a posteroanterior chest radiograph. There have been reports indicating that the use of ultrasonography guidance during CVC placement can help minimize complications [10,11,14]. In their study, Jasper et al. examined the compatibility of USG and posteroanterior chest radiograph imaging in identifying malpositions following the CVC procedure. The study’s main findings, when comparing USG with posteroanterior chest radiograph, revealed a sensitivity of 0.70 (95% CI, 0.49 to 0.86) and a specificity of 0.99 (95% CI, 0.98 to 1.00). Put simply, the outcome has been found to have a moderate level of sensitivity and a high level of specificity [15].

Although it is not very common, its placement can be confirmed by transthoracic or TEE during the procedure, and in studies where this method is preferred, TEE has been recommended to determine the guidewire position during central venous cannulation. It has been reported that anesthesiologists can detect catheter malposition at the bedside with TEE [16]. Apart from these, micro bubble test (MBT), hypertonic fluids, and echocardiographic confirmation techniques are used [17]. It is available in the literature that monitoring the central venous waveform and intravascular electrocardiogram will also be useful to confirm the position of the catheter [18-21].

It has been observed that using guides such as TEE, MBT, visualization of central venous waveforms, etc. in CVC placement, reduces the time between intervention and use [5].

Complaints such as newly developed hypotension, catheter malfunction, new chest pain if the patient is awake, back pain, and dyspnea should lead us to evaluate for malposition. If possible, the location should be confirmed in three dimensions with computed tomography (CT) [22].

## Conclusions

It is extremely important to verify the position of the CVC during and after the CVC procedure. It should not be forgotten that the CVC procedure, which is frequently used in intensive care units, is a very important procedure that requires appropriate patient positioning, clinician experience, confirmation with imaging methods, and close clinical monitoring.
